# The Bcl-2 inhibitor Obatoclax overcomes resistance to histone deacetylase inhibitors SAHA and LBH589 as radiosensitizers in patient-derived glioblastoma stem-like cells

**DOI:** 10.18632/genesandcancer.42

**Published:** 2014-11

**Authors:** Lotte M.E. Berghauser Pont, Jochem K.H. Spoor, Subramanian Venkatesan, Sigrid Swagemakers, Jenneke J. Kloezeman, Clemens M.F. Dirven, Peter J. van der Spek, Martine L.M. Lamfers, Sieger Leenstra

**Affiliations:** ^1^ Department of Neurosurgery, Brain Tumor Center, Erasmus MC, Rotterdam, The Netherlands; ^2^ Department of Bio-informatics, Erasmus MC, Rotterdam, The Netherlands; ^3^ Department of Neurosurgery, Elisabeth Medical Hospital, Tilburg, The Netherlands

**Keywords:** Bcl-2, Bcl-XL, HDAC inhibitor, SAHA, LBH589, radiation, Obatoclax

## Abstract

Glioblastoma has shown resistance to histone deacetylase inhibitors (HDACi) as radiosensitizers in cultures with Bcl-XL over-expression. We study the efficacy of SAHA/RTx and LBH589/RTx when manipulating Bcl-2 family proteins using the Bcl-2 inhibitor Obatoclax in patient-derived glioblastoma stem-like cell (GSC) cultures. GSC cultures in general have a deletion in phosphatase and tensin homolog (PTEN). Synergy was determined by the Chou Talalay method. The effects on apoptosis and autophagy were studied by measuring caspase-3/7, Bcl-XL, Mcl-1 and LC3BI/II proteins. The relation between treatment response and O6-methylguanine-DNA methyltransferase (MGMT) promoter methylation status, recurrence and gene expression levels of the tumors were studied. Obatoclax synergized with SAHA and LBH589 and sensitized cells to HDACi/RTx. Over 50% of GSC cultures were responsive to Obatoclax with either single agent. Combined with HDACi/RTx treatment, Obatoclax increased caspase-3/7 and inhibited Bcl-2 family proteins Bcl-XL and Mcl-1 more effectively than other treatments. Genes predictive for treatment response were identified, including the F-box/WD repeat-containing protein-7, which was previously related to Bcl-2 inhibition and HDACi sensitivity. We emphasize the functional relation between Bcl-2 proteins and radiosensitization by HDACi and provide a target for increasing responsiveness in glioblastoma by using the Bcl-2 inhibitor Obatoclax.

## INTRODUCTION

Patients with glioblastoma have a poor prognosis. [1, 2] Thus far, maximally safe surgical resection and radiation therapy (RTx) with adjuvant temozolomide (TMZ) gives a median survival of 14.7 months.[3] The O6-Methylguanine-DNA methyltransferase (MGMT) promoter methylation status is the most important biomarker that predicts response to current therapy. [4] Some of the causes of poor outcome lie in the heterogeneity of the tumor and the multiple escape pathways glioblastoma harbors.[5, 6] Specifically targeting these resistance pathways using combination therapies can be an approach to achieve better treatment results. A novel therapy in glioblastoma is the epigenetic modulatory treatment with histone deacetylase inhibitors (HDACi). These drugs affect multiple pathways including apoptosis, autophagy and DNA damage repair.[7] We reported earlier that a subset of patient-derived glioblastoma stem-like cell cultures (GSC cultures) showed resistance to the combination of RTx with the HDACi SAHA or LBH589. [8] Both over-expression of Bcl-XL proteins as well as the maintained Bcl-2 proteins post-HDACi treatment were related to this resistance.[8] Thus, regulation of Bcl-2 family members may be an important mechanism in the resistance to HDACi as radiosensitizers. Others have already shown that these proteins are related to resistance to HDACi as single agents.[9] The Bcl-2 family members regulates the intrinsic cell death pathway by controlling permeability of the outer mitochondrial membrane.[10] In addition, by binding the endoplasmic reticulum, these proteins regulate autophagy by binding to Beclin-1.[11] Inhibiting these Bcl-2 family proteins may provide an effective strategy in overcoming resistance to HDACi.[12] A recent study has shown efficacy of the Bcl-2 inhibitor ABT-737 and SAHA in an immortalized glioma model. Efficacy was only observed in cell lines with an intact phosphatase and tensin homolog (PTEN) status. In our study, we aim to inhibit the anti-apoptotic Bcl-2 pathway by using Obatoclax, to enhance the efficacy of SAHA and LBH589 as both single agents and as radiosensitizers in the PTEN deleted patient-derived GSC model.[13, 14] Obatoclax is a Bcl-2 family inhibitor which has higher affinity than ABT-737 to inhibit Bcl-XL, Mcl-1 and to induce autophagy.[12, 15, 16] Obatoclax reduces Bak/Mcl-1-binding, up-regulates Bim and subsequently induces cytochrome-C release and caspase-3-activity. [17] The drug is currently being tested in clinical trials for hematological cancers, non-small cell lung carcinoma and extensive-stage small-cell lung carcinoma.[18-21] Our study provides novel insights into the efficacy of inhibiting the Bcl-2 family pathway by Obatoclax in overcoming resistance to HDACi as radiosensitizers. In addition, we identify gene expression prediction profiles for response to the various treatment modalities.

## RESULTS

### Patient-derived GSC cultures show differential sensitivity to Obatoclax

**Figure 1A-D:**
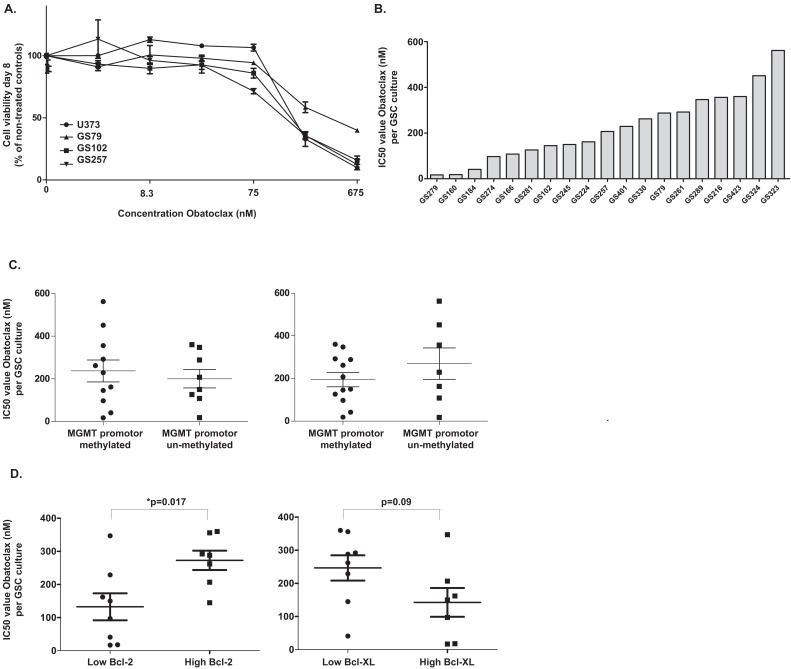
Sensitivity of patient-derived GSC cultures to Obatoclax treatment (A) The immortalized glioma cell line U373 and the GSC cultures GS102, GS79 and GS257 were incubated with a concentration-range of Obatoclax and the *IC*_50_ values were calculated based on viability on day eight by median equation. We show the viable percentage of cells compared to non-treated controls with standard errors. (B) Cells of patient-derived GSC cultures were treated with at least three different concentrations of Obatoclax in the nanomolar range. After eight days, viability was measured by CellTiter-Glo assay and the *IC*_50_ values were calculated by median equation. The graph shows the results of nineteen screened patient-derived GSC cultures. (C) MGMT promoter methylation and recurrence status of the patient-derived GSC cultures were related to the *IC*_50_ values of Obatoclax. (D) The Bcl-2 and Bcl-XL protein levels were related to *IC*_50_ values of the cultures for which this status was known.

The IC_50_ values of Obatoclax were determined in three patient-derived GSC cultures and in U373 glioma cells in order to find the right concentrations for further screening. Then, fourteen patient-derived glioblastoma cultures were screened for the combination treatments, after which validation of the results was performed in another five cultures. If the cultures were highly sensitive to Obatoclax, lower concentrations (10 and 30 nM) were used. Since the MGMT promoter methylation status is the most established predictive marker of glioblastoma survival[4], we evaluated whether treatment efficacy was related to this factor. Also we investigated the relation of response with recurrence status. Also, the relation between the IC_50_ values and Bcl-2/Bcl-XL protein levels was investigated (Figure 1A-D, Table 1). The results show that Obatoclax reduced viability in the nanomolar range. The IC_50_ values as approached by median effect equation, ranged from 17 – 562nM, varying per GSC culture. In total, eleven of nineteen GSC cultures had a methylated MGMT promoter status and seven cultures were derived from recurrent tumors. Neither of these parameters correlated to the IC_50_ values of Obatoclax (p<0.05). The protein levels of these cultures were retrieved from previous research.[8] The protein levels were categorized as high or low. In cultures with high levels of Bcl-2, the mean IC_50_ value is significantly higher than in other cultures (p=0.017). For Bcl-XL, we observed a trend that higher IC_50_ values occurred in cultures with lower Bcl-XL protein levels (p=0.09).

**Table 1:** The characteristics and responses of the studied patient-derived glioblastoma cultures

			Obatoclax	SAHA/Obatoclax		LBH589/Obatoclax		Obatoclax /RTx	
Glioblastoma culture	MGMT promoter	Recurrence	IC_50_ (nM)	Enhancement Factor	Effect Size	Enhancement Factor	Effect Size	Enhancement Factor	Effect Size
GS102	M	No	145	1.56	20.25%	1.26	0.36%	1.69	23.80%
GS160	UM	No	18	1.51	19.68%	1.33	5.61%	1.43	23.36%
GS166	UM	Yes	108	1.4	8.07%	1.5	18.05%	1.25	15.67%
GS184	M	No	41	1.85	20.80%	1.77	26.10%	1.58	15.09%
GS216	M	Yes	356	2.05	21.46%	1.1	7.25%	1.89	19.74%
GS224	M	Yes	162	2.06	20.62%	1.74	17.07%	1.07	3.59%
GS245	UM	No	150	2.23	13.65%	1.39	6.93%	1.31	5.89%
GS257	UM	No	207	2.34	20.23%	1.3	8.24%	1.8	14.07%
GS261	M	No	292	14.3	21.39%	1.17	11.97%	3.61	16.63%
GS274	M	No	97	2.26	13.30%	1.49	7.86%	2	11.93%
GS279	M	Yes	17	1.41	13.93%	1.15	10.12%	1.59	26.24%
GS281	UM	No	126	1.42	14.74%	1.22	8.97%	0.91	−4.98%
GS289	UM	No	347	2.64	20.29%	1	−0.12%	2.82	21.08%
GS323	M	Yes	562	1.16	6.34%	1.19	7.65%	1.24	12.95%
GS324	M	Yes	451	1.17	8.56%	0.92	−6.09%	1.14	8.65%
GS330	M	No	262	1.29	4.78%	1.11	9.77%	1.19	13.40%
GS401	M	Yes	229	1.21	8.12%	1.34	16.99%	1.24	9.79%
GS423	UM	No	360	3.35	18.24%	1.1	8.22%	1.49	8.51%
GS79	UM	No	288	2.74	34.54%	4.88	26.08%	1.45	10.25%

The relevant characteristics of the GSC cultures are provided, being the MGMT promoter methylation status, gender and whether or not the GSC culture was obtained from a patient with a recurrent tumor. The combination of treatments of Obatoclax with either SAHA, LBH589 or RTx (3Gy), gave differetial responses in different patient-derived GSC cultures. Red = non-responsive to the proposed treatment; blue = responsive to the proposed treatment.

### Obatoclax synergizes with HDAC inhibitors culture-dependently in patient-derived GSC cultures

**Figure 2A-C:**
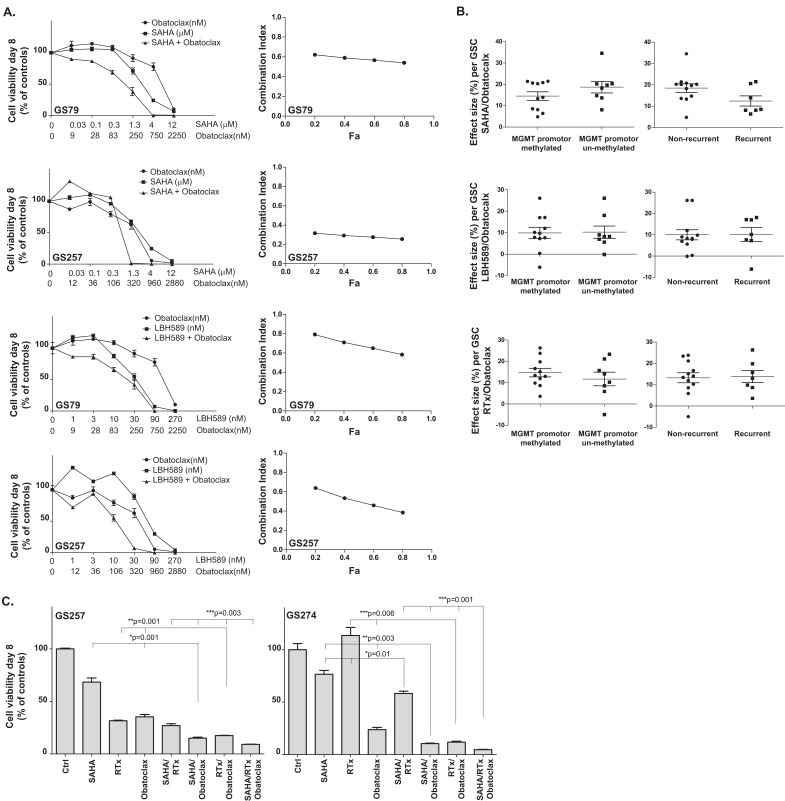
The effect of Obatoclax on HDACi and radiation treatment in patient-derived GSC cultures (A) Left: Concentration-range assays were performed to determine synergy between Obatoclax and the HDACi SAHA and LBH589 on patient-derived GSC culture GS79 (sensitive to HDACi/RTx treatment) and GS257 (resistant to HDACi/RTx). After eight days of incubation, the viability and synergy were determined by CellTiter-Glo assay and median equation calculation. Means of triplicate tests were shown with the standard errors. Right: The combination indices corresponding to the viability graphs in Figure 2A were calculated according to the Chou Talalay method determining synergism between the two drugs. Synergistic effects were observed in the two patient-derived GSC cultures for both HDACi and Obatoclax, with combination indices (CI) lower than 1 (CI<1 is considered as synergy, CI=1 is considered additive effects, CI>1 is considered as antagonism). (B) The MGMT promoter methylation and recurrence status of the patient-derived GSC cultures were related to the effect sizes of the SAHA/Obatoclax, LBH589/Obatoclax or RTx/Obatoclax treatments. No significant relations were found between responses to treatment and those important prognostic parameters. (C) The results on viability at day eight, of two patient-derived GSC cultures treated with Obatoclax combined with SAHA or LBH589, RTx and all possible combinations. RTx = radiation. * Indicates significance at p<0.05 for the different combination treatments compared to as indicated in the graphs by the vertical lines.

**Figure 3A-C:**
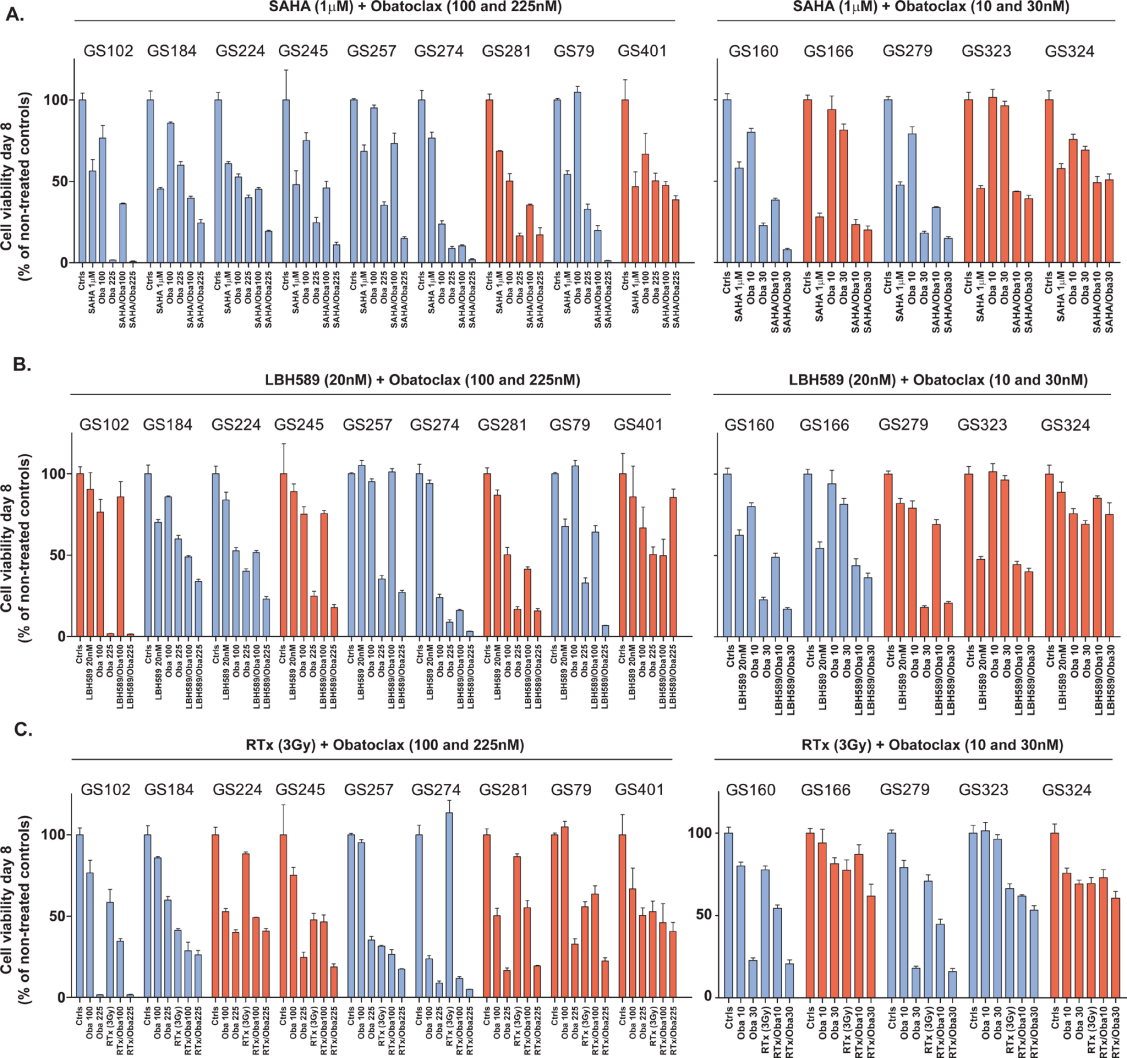
The effects of Obatoclax on HDACi in patient-derived GSC cultures (A) Nine (left, high concentrations) and five (right, low concentrations) patient-derived GSC cultures were treated with two different concentrations of Obatoclax and SAHA (1μM). After eight days viability was measured by CellTiter-Glo assay. Responders (green bars) and non-responders (red bars) were identified. The color blue indicates significance at the p<0.05 level for one of the concentrations. (B) Nine (left, high concentrations) and five (right, low concentrations) patient-derived GSC cultures were treated with two different concentrations of Obatoclax and LBH589 (20nM). After eight days viability was measured by CellTiter-Glo assay. Responders (green bars) and non-responders (red bars) were identified. The color blue indicates significance at the p<0.05 level. (C) Nine (left, high concentrations) and five (right, low concentrations) patient-derived glioblastoma cultures were treated with two different concentrations of Obatoclax (Oba) and RTx (3Gy). After eight days viability was measured by CellTiter-Glo assay. Responders (green bars) and non-responders (red bars) were identified. The color blue indicates significance at the p<0.05 level.

To determine synergy between Obatoclax and the HDACi SAHA or LBH589, the Chou Talalay method was used in the patient-derived GSC cultures GS79 and GS257. Next, the panel of fourteen and five patient-derived GSC cultures were screened with the previously mentioned concentrations of Obatoclax, combined with SAHA and LBH589, and with or without RTx. Also, the MGMT promoter methylation and recurrence status of the tumors were related to responses to treatment. For both cultures GS79 and GS257, Obatoclax showed synergistic effects with SAHA and LBH589 with combination indices < 1 (Figure 2A). The combination efficacy widely varied in the panel of patient-derived GSC cultures: responders and non-responders were identified for all combination treatments (Table 1, Figure 3). In the first set of glioblastoma cultures (n=14) eight cultures responded to SAHA/Obatoclax treatment. In the validation set (n=5) four cultures responded to treatment. Enhancement factors ranged from 1.2 – 14.3 with effect sizes ranging from 5% - 35%. Seven of the fourteen cultures responded to LBH589/Obatoclax treatment and three of the five cultures in the validation set responded to treatment. The enhancement factors ranged from 0.9 – 4.9 with effect sizes of −6% – 26%. Obatoclax sensitized seven of fourteen GSC cultures to radiation, and all the GSC cultures in the validation set responded to this treatment. The enhancement factors ranged from 0.9–3.6 with effect sizes ranging from −5% – 26%. The MGMT promoter methylation and recurrence status were not related to the effect sizes of the tested combination treatments (p>0.05) (Figure 2B).

### Obatoclax overcomes resistance to HDAC inhibitors as radiosensitizers in patient-derived GSC cultures

To evaluate whether treatment with Obatoclax sensitizes patient-derived GSC cultures to HDACi as radiosensitizers, the nineteen patient-derived GSC cultures were evaluated (Figure 2C). The co-treatment with Obatoclax resulted in a significant reduction in cell viability compared to the combination treatment of RTx and SAHA without Obatoclax. However the effect sizes of triple treatment were small, since the ATP-levels at day eight show that either HDACi/Obatoclax, Obatoclax/RTx or HDACi/RTx already inhibited the largest part of the cell population. In the patient-derived GSC cultures GS257, GS274, GS224 and GS401, the effect sizes ranged from 5-9% and the enhancement factor ranged from 1.1 – 2.2 (GS257 and GS274 shown in Figure 2C). The addition of Obatoclax to the combination treatment of LBH589/RTx did not result in significant additional treatment effects.

### Obatoclax triggers apoptosis and induces autophagy in both HDAC inhibitor and HDACi/RTx combination treatment

**Figure 4A-C:**
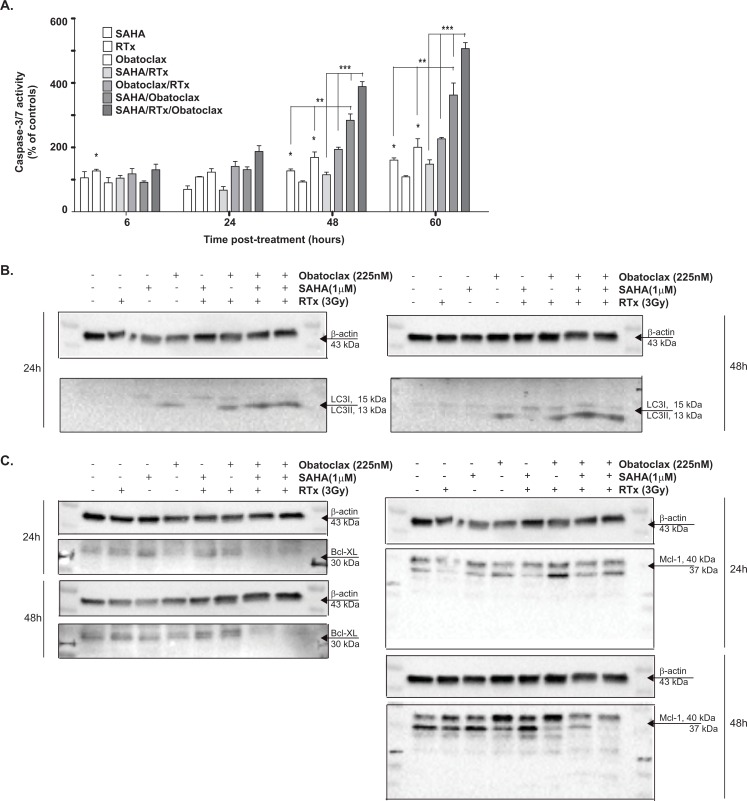
The mechanisms involved in the efficacy of the combination treatments (A) Cells of the GSC culture GS257 were treated with Obatoclax (225nM), SAHA (1μM) and all possible combinations. After 24 hours, cells were radiated or not (controls) with 3Gy. Caspase-3/7 reagent was added to the wells, the plates were placed in an IncuCyte and followed for 60 consecutive hours. Fluorescence was measured using the IncuCyte software. *Indicates significance at p<0.05 compared to non-treated controls. **/***Indicate significance at p<0.05 of the treatments, compared to the treatments as indicated by the vertical bars (compared to either 2 or 3 treatments). (B) The GSC cultures GS257 were treated as in Figure 4A. Cells were collected either 24 or 48 hours post-treatment with RTx. The conversion of LC3BI/II, indicating autophagy was studied by using the Western blot analysis. (C) The cells of the GSC culture GS257 were treated as in Figure 4A. Cells were collected either 24 or 48 hours post-treatment with RTx. The anti-apoptotic Bcl-2 family proteins Bcl-Xl and Mcl-1 were studied by using the Western blot analysis.

The CellTiter-Glo assay at day eight may have underestimated the effects of triple treatment due to the lack of differentiation between the effective treatments at this time point. We hypothesized that early treatment differences could be observed by studying the important cell death mechanisms of the Bcl-2 pathway: apoptosis and autophagy.[22] We studied early caspase-3/7 activation, which could provide more insights into the difference in efficacy between triple and combination treatment. The activated caspase-3/7 levels were monitored over time for SAHA concentrations at which additional effects were observed after triple treatment in the viability assay. The HDACi SAHA and the Bcl-2 family inhibitor Obatoclax activated the caspase-3/7 activity at both 48 and 60 hours post-treatment (Figure 4A). There was no additional caspase-3/7 activation in case of the combination treatment of SAHA and RTx, in comparison to single agents alone. Combination treatment of RTx/Obatoclax did not increase caspase-3/7 activity in comparison to single agent treatment either. In both SAHA/Obatoclax and SAHA/Obatoclax/RTx treated cells, increased caspase-3/7 activity was observed at 48 and 60 hours (p<0.05), with SAHA/Obatoclax/RTx being significantly more effective than SAHA/Obatoclax (p<0.05). As well as inducing caspase-dependent apoptosis, the Bcl-2 family proteins play a crucial role in autophagy and autophagosome formation which can be studied by conversion of LC3BI/II proteins. Treatment of GS257 cells with either SAHA, RTx and SAHA/RTx did not result in conversion of LC3I/LC3II at 48 and 72 hours post-treatment (Figure 4B). On the contrary, treatment with Obatoclax strongly induced autophagy at 48 hours post-treatment. Obatoclax increased both LC3I and LC3II in combination with RTx. In the SAHA/Obatoclax and Obatoclax/SAHA/RTx treated cells, LC3BI/II conversion (LC3II divided by LC3I) was observed at both 48 and 72 hours post-treatment. The triple treatment showed a higher ratio of LC3BI/II conversion.

### Obatoclax combined with HDACi and HDACi/RTx effectively decreases Bcl-XL and Mcl-1

As increased autophagy and apoptosis were observed in both the combination and triple treatment, we related these effects to altered levels of the anti-apoptotic Bcl-2 proteins Bcl-XL and Mcl-1 in the patient-derived glioblastoma culture GS257 (Figure 4C). The protein levels of Bcl-XL were decreased by Obatoclax at 48 hours post-treatment, but after 72 hours the levels were similar to controls. The HDACi SAHA even induced Bcl-XL protein levels at 48 hours, whereas at 72 hours, these levels were again similar to controls. The combination treatments of SAHA/RTx and Obatoclax/RTx did not affect Bcl-XL protein levels, whereas SAHA/Obatoclax and SAHA/Obatoclax/RTx completely eliminated Bcl-XL protein levels after both 48 and 72 hours. For Mcl-1, the most important effects were observed 48 hours after RTx: the levels were decreased by Obatoclax/RTx, Obatoclax/SAHA and to a larger extent by Obatoclax/SAHA/RTx. All these treatments induced additional Mcl-1 bands on the Western blot at both 37kDa and 25 kDa. The Mcl-1 protein levels (37 and 40 kDa) of cells treated by the latter two treatments were relatively low compared to the other combination treatments at 72 hours. In addition, the two additional Mcl-1 bands were induced by these treatments at both 15 and 17 kDa. These bands may correspond to cleaved Mcl-1, which has been associated with activation of apoptosis.

### Levels of specific genes correlate with response of GSC cultures to combination treatments with Obatoclax

**Figure 5A-E:**
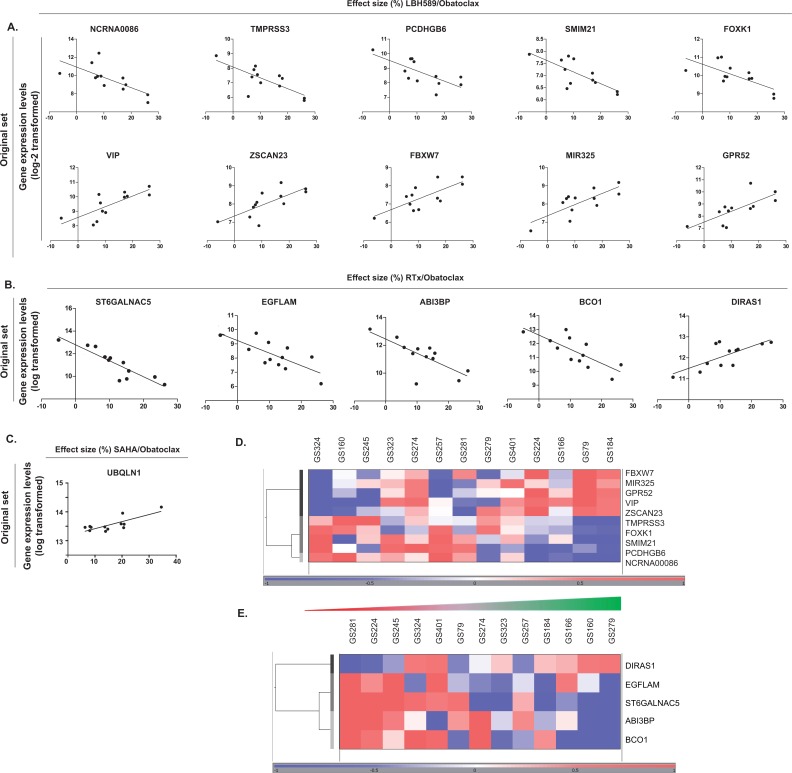
Gene expression profiling of the response to treatments with Obatoclax (A) The gene expression levels that were associated with the effect sizes to LBH589/Obatoclax treatment. Criteria were a p-value of <0.05, and a correlation coefficient of >0.5 or <-0.5. The gene expression intensity levels are displayed on log-2 scale. (B) The gene expression levels that are associated with the effect sizes to RTx/Obatoclax treatment. Criteria were a p-value of <0.05, and a correlation coefficient of >0.5 or <-0.5. The gene expression intensity levels are displayed on log-2 scale. (C) The gene expression levels that are associated with the effect sizes to SAHA/Obatoclax treatment. Criteria were a p-value of <0.05, and a correlation coefficient of >0.5 or <-0.5. The gene expression intensity levels are displayed on log-2 scale. (D,E) Heat map of the genes that correlated to the effect sizes of the combination treatments of LBH589/Obatoclax, RTx/Obatoclax and SAHA/Obatoclax Gene expression levels: red, up-regulated genes compared with the geometric mean; blue, down-regulated genes compared with the geometric mean. The color intensity correlates with the degree of change. The green-to-red intensity bar correlates with the degree of change in effect sizes of the corresponding GSC cultures based on the viability assays.

In order to identify predictive markers for treatment response, the gene expression levels of the original tumor were related the treatment response of the fourteen patient-derived GSC cultures. The quality assessment of the gene expression array data showed that data of thirteen samples could be used for the analysis (GS102 was excluded). The validation of the identified genes that correlated with treatment response was performed in an independent set of five patient-derived GSC cultures. This resulted in a specific set of genes of which levels were related to treatment response in both the initial and validation set. For selection, at least a 1.5-fold change (log-2 scale) was the minimum difference in gene expression between the most and the least responsive patient-derived glioblastoma culture. The functions of the identified genes were analyzed using the QIAGEN's Ingenuity® Pathway Analysis. We focused on the functions and networks of the genes which were associated with the combination responses. Table 2A shows the gene lists per treatment, the correlation coefficient with the treatment effects and the fold-change difference in gene expression levels between the most and least sensitive corresponding glioblastoma culture. The responses to LBH589/Obatoclax treatment were related to gene expression levels of ten genes, being the non-coding RNA NCRNA00086, transmembrane protease serine 3 (TMPRSS3), proto-cadherin gamma-subfamily B6 (PCDHGB6), small integral membrane protein 21 (LOC284274 or SMIM21), forkhead box K1 (LOC642782 or FOXK1), vasoactive intestinal peptide (VIP), zinc finger and SCAN domain containing 23 (ZSCAN23), F-box and WD repeat domain containing 7, E3 ubiquitin protein ligase (FBXW7), microRNA 325 (MIR325), and G protein-coupled receptor 52 (GPR52) (Figure 5A,D). This set genes were found to be functioning in processes of cell cycle (FBXW7, VIP, FOXK1), cell death and survival (VIP, FBXW7, PCDHGB6) and cell morphology (FBXW7, VIP) as top three gene functions (Table 2B). Another group recently showed that mutations in FBXW7 were related to SAHA-induced cell death and Bcl-2 inhibition.[12] The genes that were related to responses to Obatoclax/RTx treatment were ST6 (alpha-N-acetyl-neuraminyl-2,3-beta-galactosyl-1,3)-N-acetylgalactosaminide alpha-2,6-sialyltransferase 5 (ST6GALNAC5), EGF-like, fibronectin type III and laminin G domains (EGFLAM), ABI family member 3 (NESH) binding protein (ABI3BP), beta-carotene oxygenase 1 (BCO1) and DIRAS family GTP-binding RAS-like 1 (DIRAS1) (Figure 5B,E). These genes were involved in cellular development and cellular growth and proliferation (DIRAS1), as well as lipid metabolism (BCO1, ST6GALNAC5). Moreover, all the five genes were found to function in the same network, namely a cell cycle network (Table 2B). The responses to SAHA/Obatoclax treatment were related to one gene, namely ubiquilin 1 (UBQLN1) (Figure 5C). This gene is involved in cell morphology, cellular compromise and cell death and survival according to the functional analysis. In summary, we present a number of genes that is related to the responses of patient-derived GSC cultures to combination treatments with Obatoclax, RTx and HDACi. Functional analysis showed that most of these genes were related to cell regulatory aspects including cell death and survival, as well as cycle regulatory processes.

**Table 2A:** Genes that are related to the response to Obatoclax combination treatments *in vitro*

	Gene		Corr.	Chrom. Locus	Least sensitive	Most sensitive	FC (log)
**Treatment: LBH589/Obatoclax**							
1	NCRNA00086	long intergenic non-protein coding RNA 86	−0.70	X	7.02	10.23	3.21
2	TMPRSS3	transmembrane protease, serine 3	−0.73	21	5.94	8.86	2.92
3	PCDHGB6	protocadherin gamma subfamily B, 6	−0.69	5	7.86	10.26	2.40
4	LOC284274	SMIM21 small integral membrane protein 21	−0.71	18	6.21	7.87	1.66
5	LOC642782		−0.71		8.74	10.28	1.53
6	VIP	vasoactive intestinal polypeptide	0.69	6	10.12	8.53	−1.59
7	ZSCAN23	zinc finger and SCAN domain containing 23	0.71	6	8.67	7.02	−1.65
8	FBXW7	F-box and WD repeat domain containing 7, E3 ubiquitin protein ligase	0.71		8.09	6.22	−1.87
9	MIR325	microRNA 325	0.70	X	8.55	6.52	−2.03
10	GPR52	G protein-coupled receptor 52	0.70	1	9.29	7.14	−2.14
**SAHA/Obatoclax**							
1	UBQLN1	ubiquilin 1	0.78	9	14.17	13.00	−1.16
**Radiation/Obatoclax**							
1	ST6GALNAC5	ST6 (alpha-N-acetyl-neuraminyl-2,3-beta-galactosyl-l,3)-N-acetylgalactosaminide alpha-2,6-sialyltransferase 5	−0.74	1	9.27	13.21	3.94
2	EGFLAM	EGF-like, fibronectin type IIIand laminin G domains	−0.72	5	6.19	9.60	3.42
3	ABI3BP	ABI family, member 3 (NESH)binding protein	−0.70	3	10.16	13.16	3.00
4	BCMOl	beta-carotene 15,15′-monooxygenase	−0.72	16	10.47	12.83	2.36
5	DIRAS1	DIRAS family, GTP-bindingRAS-like 1	0.73	19	12.75	11.08	−1.67

**Table 2B:** The top three functions and networks of the genes that are related to the response to Obatoclax combination treatments *in vitro*

**LBH589/Obatoclax**	
**Function**	**Gene**
Cell Cyle	FBXW7, VIP, FOXK1
Cell Death and survival	VIP, FBXW7, PCDHGB6
Cell Morphology	FBXW7, VIP
**SAHA/Obatoclax**	
**Function**	**Gene**
Cell Morphology	UBQLN1
Cellular Compromise	UBQLN1
Cell Death and Survival	UBQLN1
**RTx/Obatoclax**	
**Function**	**Gene**
Cellular Development	DIRAS1
Cellular Growth and Proliferation	DIRAS1
Lipid Metabolism	BCOl, ST6GALNAC5
Cell Cycle*	ABI3BP, BCOl, DIRAS1, EGFLAM, ST6GALNAC5

(A) The gene expression levels that were associated with the effect sizes of the several treatments with Obatoclax. The criteria were p-value of <0.05, a correlation coefficient of >0.5 or <-0.5. The gene expression intensity levels are displayed on log-2 scale. FC = fold change between the least and most sensitive patient-derived GSC cultures.

(B) The functions and networks of the specific genes derived from the Ingenuity Pathway Analysis.

## DISCUSSION

The present study emphasizes the efficacy of the Bcl-2 family pathway inhibition by Obatoclax in sensitizing patient-derived GSC cultures to HDACi/RTx, and hereby provides a circumvention for a tumor-related resistance mechanism to treatment. The Bcl-2 family proteins are heterogeneously expressed in glioblastoma as about 30%[23] to 60%[24] shows over-representation of these proteins.[24-26] Moreover, Bcl-XL levels in patient-derived GSC cultures have shown a relationship with resistance to HDACi/RTx response.[8] Here, we demonstrate that Obatoclax acted synergistically with HDACi and show efficacy in a large set of patient-derived glioblastoma cultures. Hereby we confirm that this pathway is an adequate target to obtain treatment responses. The use of the serum-free patient-derived GSC culture model allowed to study resistance and tumor response mechanisms in a more representative manner than in immortalized glioma cell lines.[13] Importantly, this model provides a tool to develop predictive profiles of treatment response. The efficacy of down-regulation of Bcl-2 and Bcl-XL in inducing spontaneous cell death was already shown before.[27] This efficacy depended on neither MGMT promoter methylation status nor recurrence. A number of genes was found to be related to treatment response of patient-derived GSC cultures to combination treatments. Others showed that the gene FBXW7 was related to SAHA-induced cell death and Bcl-2 inhibition.[12] This points to a biological rationale in Mcl-1 functioning to be predictive of treatment response to LBH589/Obatoclax. The use of these genes as potential markers for patient stratification or the functionality of these genes in sensitivity to combination treatments should be explored in future studies.

Others have studied the relationship between HDACi and Bcl-2 family proteins before. A recent study has shown that the intact PTEN immortalized malignant glioma cell lines are susceptible to the combination of SAHA and the Bcl-2 inhibitors ABT-737, whereas PTEN mutations were not.[28] They also showed that inhibition of the PTEN/PI3K pathway by BKM-120 sensitized glioma cells for ABT-737.[29] The patient-derived GSC model we used in this study is known to harbor PTEN deletions due to loss of chromosome 10q [13, 14], and ensures that we tested the treatment efficacy in a setting of hyper-activation of the PIK3-pathway. Therefore, this suggests that Obatoclax is a more potent combination partner for HDACi in PTEN deleted tumors than ABT-737. The main differences between Obatoclax and ABT-737 are the stronger induction of autophagy[16] and the stronger inhibition of Mcl-1 by the first drug. Both can be underlying the efficacy in PTEN deleted tumors.[15] Earlier, the efficacy of SAHA was related to Mcl-1 levels in acute myeloid leukemia.[30] Another study showed that in diffuse large B-cell lymphoma, over-expression of Bcl-2 and Bcl-XL triggered resistance to the HDACi SAHA and Trichostatin A, whereas the Bcl-2 family inhibitor ABT-737 increased sensitivity to SAHA treatment. Also, this study showed that the least sensitive cells to SAHA showed up-regulation of Bcl-XL and Mcl-1 proteins.[31] The loss of pro-apoptotic Bcl-2 family proteins Bax or Bax/Bak ratios are related to insensitivity to HDACi in Burkitt's lymphoma in a predictive and functional sense. [32] In mantle cell lymphoma, the efficacy of SAHA was enhanced by the BH-3 mimetic ABT-263.[33] In contrast, another study did not find HDACi efficacy to be related to Bcl-2 family proteins.[31] Thus, these relationships between Bcl-2 family proteins and HDACi responses are either tumor type or HDACi specific. Our study shows that inhibition of the Bcl-XL and Mcl-1 proteins by Obatoclax is related to efficacy of SAHA alone or in combination with RTx. We observed restored levels of Bcl-2 family proteins Bcl-XL and Mcl-1, occurring at 72 hours after Obatoclax, HDACi and HDACi/RTx treatments. This observation indicates that Bcl-2 family members are restored within this time frame, if these are not inhibited effectively. This in turn can contribute to resistance to treatment.

As a mechanism of action, HDACi have shown to be able to down-regulate Bcl-2 family members, however not in all heterogeneous tumors including glioblastoma. [34] Others suggest that the up-regulation of pro-apoptotic members by HDACi are neutralized by high levels of anti-apoptotic members such as Bcl-2 and Bcl-XL.[35] In squamous cell carcinoma Mcl-1 was related to resistance to the BH3-mimetic ABT-737. The HDACi SAHA has the ability to synergize with this drug by shuttling the Bcl-2 family protein Bim from Mcl-1 to Bcl-2/Bcl-XL.[12]

Not only does Obatoclax enhance the efficacy of HDACi/RTx, it also enhanced the efficacy of RTx as treatment alone. Thus, in many treatment modalities, including conventional glioblastoma treatment such as RTx, the Bcl-2 family members may play a crucial role. Others have shown already that Bcl-2 family members are determinants of responsiveness to treatment in glioblastoma. Bcl-XL levels were related to radioresistance in glioma [36] and Mcl-1 proteins regulate temozolomide efficacy in glioma independently of MGMT promoter methylation status.[37] Here, we found that MGMT promoter methylation status and tumor recurrence, which normally determine patient overall outcome[4], were not related to the applied treatments either. Thus, our results are unlikely to be determined by these tumor statuses *a priori*.

The effect sizes of both SAHA and LBH589 in combination with Obatoclax correlated to expression levels of specific genes. Gene profiles may provide an opportunity to include predictive decision variables in treatment and patient selection. This may be of clinical relevance specifically for the MGMT un-methylated tumors. We need to point out immediately that these gene sets need further exploration in *in vivo* settings to be validated. Although these gene sets do not necessarily implicate mechanisms of action of the proposed treatments, they may provide insight into resistance mechanisms. FBXW7, the gene associated with LBH589/Obatoclax response, was shown to have a relationship with response to SAHA before, as well as to Bcl-2 inhibition. Both FBXW7 and Bcl-2 inhibition were predictive for efficacy of SAHA in squamous cell carcinoma. Mutated FBXW7 sensitized cells to HDACi and stabilized Mcl-1.[12] FBXW7 encodes for the protein ubiquitin protein ligase and is positively related with the effect size of LBH589/Obatoclax in our study. Obatoclax has the potency to inhibit Mcl-1, and is more effective in combination with LBH589 in tumors that highly express FBXW7. Moreover, the whole gene set which was related to LBH589/Obatoclax response was associated with cell cycle and cell death and survival mechanisms, represented by the four genes FBXW7, VIP, FOXK1 and PCDHGB6. This was also true for the gene ubiquilin 1 (UBQLN1), which was associated to SAHA/Obatoclax response. This gene functions in cell death and survival as well. In addition, response to RTx/Obatoclax was associated to genes that function in the cell cycle and cellular proliferation as well. Basic studies should be performed to assess the direct relationship between the function of these genes and the success rate of Obatoclax, but the genes provide insights into the process of cellular responses. Our data on cell regulation by caspase-3/7 and autophagy are therefore potentially important mechanisms of action in the response to combination treatment.

In summary, this study underlines the potential of inhibiting the Bcl-2 family proteins in combination treatment with HDACi and HDACi/RTx. Potential toxicities of combination therapies which may limit clinical use should be explored further. Examples could be thrombocytopenia by inhibition of Bcl-XL[38] and HDACi.[39] Furthermore, we obtained predictive gene profiles that are associated with cellular regulatory functions for the combination treatments with Obatoclax. These gene sets may aid in selecting the tumors most responsive to treatment.

## MATERIALS & METHODS

### Chemicals

Stocks of 50mM SAHA (Cayman chemicals, MI, USA), 200μM LBH589 (Biovision, CA, USA) and 60mM Obatoclax (Selleck Chemicals, Texas, USA) were prepared in dimethyl sulfoxide (DMSO, Sigma-Aldrich, MO, USA) and stored at −20°C. Staurosporin was obtained from BioMol, Germany.

### Patient-derived glioblastoma stem-like cell cultures

Fresh glioblastoma tissue was obtained from patients undergoing surgery at the Department of Neurosurgery, ErasmusMC (Rotterdam, The Netherlands) after informed consent and approval by the institution's medical ethical board. The tissue was dissociated mechanically and enzymatically, after which the patient-derived GSC cultures were cultured under serum-free conditions in DMEM/F12 medium supplemented with 2% B27 (Life Technologies, UK), 20ng/ml bFGF, 20ng/ml EGF (Tebu-Bio, France), 5ug/ml heparin (Sigma-Aldrich, MO, USA) as was described previously.[13] These patient-derived GSC cultures, which were characterized and validated as previously reported.[13] The U373 cells were cultures under 10% serum conditions in DMEM medium. The cultures were stored at 37 °C in a humid 95% air/5% CO_2_ chamber. In total, nineteen patient-derived GSC cultures were used for the experiments.

### Viability assay

Concentration-response assays were performed in order to determine *IC*_50_ values of Obatoclax. The cells of various GSC cultures were plated at 1×10^3^ cells/well in 96-wells plates. After incubation overnight, various drug concentrations in three-fold increments were applied to the cells. At five and eight days post-treatment cell viability was measured using the CellTiter-Glo assay (Promega, WI, USA). The results were plotted and the IC_50_ values were computed using the median effect equation.[41] The *IC*_50_ values of the HDACi SAHA and LBH59 in the GSC cultures GS79 and GS257 were derived from previous research.[8] Subsequently, Chou-Talalay assays were performed on the GSC cultures GS79 and GS257 for Obatoclax in combination with the HDACi to determine synergy.[40] After assessment of the combination effects in these two cultures, either two or four concentrations (10, 30, 100 and 225nM) were applied to the panel of patient-derived GSC cultures to determine the combination effects. From these concentrations, an estimated *IC*_50_ was calculated for each glioblastoma culture. The HDACi concentrations were always kept similar at 1μM SAHA and 20nM LBH589. The DMSO concentration was never above 1% in the dilutions. RTx (3Gy) was applied as single-fraction treatment from a Cesium-137 source 24 hours after drug application.

### Western blot

Alteration of specific proteins post-treatment was determined by seeding cells of the GSC culture GS257 in culture flasks with 225nM Obatoclax, 1 μM SAHA and the combination. After 24 hours, the cells were irradiated with 3Gy. At 24 and 48 hours post-RTx, the cells were harvested, washed with PBS and collected in a 1% Triton-X100 (Sigma-Aldrich) buffer for protein isolation. Protein concentrations were measured with the BCA Protein Assay Reagent Kit (Thermo Fisher Scientific, MA, USA). Protein separation was performed on a 4-15% pre-casted gel (Bio-Rad, CA, USA), and blotted onto a PVDF membrane (Immobilon-P, Millipore, MA, USA) using the Mini-Protean Tetra Cell system (Bio-Rad). After blocking the membranes with 5% non-fatty milk solution for 30 minutes at room temperature, the blots were incubated with primary antibodies against Bcl-XL, LC3BI/II, Mcl-1 (1:375, Cell Signalling, MA, USA) and anti-β-actin (1:5,000, Millipore) in 5% BSA/TBS-T overnight. Membranes were washed with TBS-T 0.2% and incubated with secondary antibodies, anti-rabbit-HRP and anti-mouse-HRP (1:2000, Dako Denmark A/S, Denmark) 1½h at room temperature and detected by chemiluminescence using the Pierce ECL substrate (Thermo Fisher Scientific) and the ChemiDoc MP system (BioRad). The data were analyzed using the ImageLab software (Bio-rad).

### Caspase-3/7 assay

The GS257 GSC cultures were seeded at 5×10^3^ cells/well in a black 96-wells plate and incubated with the treatment as performed in the viability and Western blot experiments (SAHA, Obatoclax, after 24 hours RTx). Immediately after RTx, the cells were incubated with 5μM of the kinetic apoptosis reagent of the CellPlayer 96-Well Kinetic Caspase-3/7 Apoptosis Assay (Essen Bioscience) and were live imaged by using the IncuCyte system (Essen BioScience) with a 10X objective at 37°C in a humid 95% air/5% CO_2_ chamber. The apoptosis-inducer staurosporin (20nM) was used as a positive control. Three fluorescent images/well were collected up to 60 hours post-RTx. The caspase-3/7 activity was presented as counted objects/mm^2^ as measured by the IncuCyte software.

### Gene expression analysis

The RNA of the original glioblastoma tissue corresponding with the glioblastoma cultures was isolated with the RNeasy Mini kit (#74104, Qiagen Inc., CA, USA). The mRNA expression levels of were analysed by the HumanHT-12 v4 Expression BeadChip microarray (Illumina, CA, USA). The raw intensity values of all the samples were normalized using quantile normalization with the Partek software, version 6.6 (Partek Inc., St. Louis, MO, USA). Subsequently, the Partek Batch Remover was used to remove the effect of the differences between the batches. The effect sizes of HDACi/Obatoclax, and RTx/Obatoclax in n=13 GSC cultures (mRNA expression data of GS102 did not meet quality standards) were correlated to the gene expression levels. The data were analyzed using the linear model function in R package ‘stats’ (version 2.15.3) to identify genes that correlate to treatment responses (Pearson's R correlations). The p-values were adjusted for multiple comparisons using the method of Benjamini & Hochberg. [41] The cut-off values for significantly expressed genes were the adjusted p-values (p<0.05). The initially identified genes that significantly correlated with response were validated using a sample set of five additionally treated GSC cultures (p<0.05). Heat maps of the genes were constructed by the OmniViz Treescape software. The functional and network analyses of the identified genes were performed using the QIAGEN's Ingenuity® Pathway Analysis (IPA®, QIAGEN Redwood City, www.qiagen.com/ingenuity).

### Quantitative PCR on MGMT promoter methylation

The DNA of the various patient-derived GSC cultures was isolated and 100ng was used for the quantitative polymerase chain reaction (PCR). The DNAsamples were modified with sodium bisulphite using the EZ DNA Methylation GoldTM kit (Zymo Research, Baseclear, The Netherlands) Primers specific for methylated and un-methylated MGMT promoter DNA were used as described by others.[42] Methylation specific primers were F: TTTCGACGTTCGTAGGTTTTCGC and R: GCACTCTTCCGAAAACGAAACG. The un-methylated specific primers were F: TTTGTGTTTTGATGTTTGTAGGTTTTTGT and R: AACTCCACACTCTTCCAAAAACAAAACAQ. The PCR conditions were used as described by others[43], with annealing temperature of 59°C and 40 cycles of amplification yielding PCR products for methylated and un-methylated DNA of 80 and 93 base pairs, respectively. The PCR products were separated on a routine 2% agarose gel. As a methylated control the DNA isolated from FFPE human colorectal cancer cell line SW48 was used and FFPE human tonsil DNA was used as an un-methylated control. In addition, in each experiment a H_2_O sample without DNA was used as a negative contamination control.

### Statistical analysis

For the viability and caspase-3/7 assay experiments, the means of triplicates were plotted with the standard deviations. The Western blots were performed in singlicate. The results were presented as a percentage of non-treated controls. The differences between treatment effects were analyzed using the one-way ANOVA and the Tukey Post-Test for multiple conditions. The statistical significance was defined as p<0.05. The differences in mean cell viability between combinations were presented as effect sizes. In addition, we calculated the enhancement factor of combination treatments, as was described by Chou.[44] In case of an enhancement factors > 1 in combination with p<0.05, the combination was considered effective and the GSC was defined as a responder.
